# BMC Medicine: a decade of open access medical research

**DOI:** 10.1186/1741-7015-12-4

**Published:** 2014-01-09

**Authors:** Sabina Alam, Jigisha Patel

**Affiliations:** 1 BioMed Central Ltd, 236 Gray’s Inn Road, London WC1X 8HB, UK

**Keywords:** Open access, Translational medicine, Clinical oncology, Evidence-based medicine, Reporting guidelines

## Abstract

On 24 November 2003, *BMC Medicine* published its first article. Ten years and over 900 articles later we look back at some of the most notable milestones for the journal and discuss advances and innovations in medicine over the last decade. Our editorial board members, Leslie Biesecker, Thomas Powles, Chris Del Mar, Robert Snow and David Moher, also comment on the changes they expect to see in their fields over the coming years.

## Editorial

Just a few months after the Human Genome Project was declared complete [1]*BMC Medicine* was launched as an open access [2,3], open peer review journal (i.e. where signed peer review reports are published with the article) [4,5], with the aim of making high impact clinical peer-reviewed research of general interest, accessible to everyone from the basic scientist to the practicing clinician. The journal, initially under the direction of Pritpal Tamber and then Melissa Norton as Editor-in-Chief, was launched amidst a raging debate about the viability of open access publishing [6]. But open access survived and evolved [7,8], and *BMC Medicine* now ranks 8^th^ out of 155 journals in the 2012 general and internal medical journals category of the Journal Citation Reports [9].

While mainly focused on primary research in its early days, the journal responded to the needs and demands of its readers and contributors by providing, for example, a platform for discussing controversies in medical practice [10-15] and embracing social networking technology to promote open scientific discussion and debate [16-19]. Although proud of its Impact Factor (IF) of 6.68, *BMC Medicine* recognizes that the IF is a restrictive metric that does not fully reflect the influence of individual articles post-publication. The journal therefore provides informative article metrics which are immediately available on published articles - a feature which has proven to be popular with many of our authors [20].

Of course, innovations in publishing and technical advances notwithstanding, *BMC Medicine* owes its success to the scientific contributions made by its authors, reviewers and expert editorial board members. To celebrate its 10^th^ anniversary, we recently reviewed some of our most successful articles in terms of accesses [21], citations [22] and ‘impact’ in news and social media [23], and also summarized author and reviewer experiences [24] and explored our author demographics [25]. As a general medical journal with a very broad scope, it is not possible for us to cover all the main advances in medicine featured in the journal over the last decade, but in this editorial we present a selection of our favorite recent content, together with predictions by our editorial board members on possible future directions for their respective fields of research.

## Translational medicine: how far have we come with stem cells, biomarkers and ‘Omics’ research?

Stem cell research and therapy has advanced rapidly in the last decade, and clinical trials for a wide range of diseases are already underway [26,27]. In 2012, the journal published an intriguing study by Zhao and colleagues, who used Stem Cell Educator therapy to safely reverse Type 1 diabetes. The researchers used stem cells from cord blood to ‘re-educate’ T cells in patients with Type 1 diabetes, thereby restoring pancreatic function and reducing the need for insulin [28]. These compelling results highlight how stem cell therapies may become part of mainstream treatment for many diseases.

Within the last 10 years, major advances have also been made in biomarker research and ‘Omics’ studies in a preclinical setting. Advances in whole genome sequencing have allowed the identification of genes involved in a large number of diseases, and biomarkers that indicate disease severity or susceptibility to treatment are increasingly being characterized [29-32]. As Leslie Biesecker points out (Box 1), clinical exome and genome sequencing are already being used in the clinic for diagnostic and prognostic purposes. However, Biesecker also indicates that these new technologies are not without problems and alludes to the role the journal plays in ensuring the latest research is appropriately validated and disseminated.

Despite the discovery of many biomarkers for cancer in particular, so far very few have been used within the clinical setting [33], which is partly due to a lack of consistency and clarity in the reporting of prognostic tumor markers. This prompted the development of the Reporting Recommendations for Tumor Marker Prognostic Studies (REMARK) checklist [34], which was updated in 2012 by Altman and colleagues [35] and more recently, the development of criteria to address the lack of scientific rigor when evaluating preclinical evidence to support translation of omics-based predictors to clinical trials [36,37].

The continued identification of new genes and biomarkers specific to disease subtypes and individual patients is essential for translation into personalized medicine, in terms of estimating both disease risk and response to therapy. As highlighted above, the field which has seen the most progress in this area is clinical oncology, and Thomas Powles explains what further changes are required to achieve effective personalized cancer therapies (Box 2).

## Box 1

**Leslie G. Biesecker**National Human Genome Research Institute, USA

Genomics has provided new modes of discovery in the basic sciences and is now doing the same for clinical research. It has already started to change medical practice with clinical exome and genome sequencing. These two assays are now being used in thousands of patients, for example, to provide a genome-wide diagnostic assay for uncharacterized disorders of birth defects or neurologic disorders and in tumor sequencing to identify targets for cancer therapeutics. *BMC Medicine* has a critical role to fulfil in this process by providing a forum for critical evaluation of these new technologies. The objective could not be more important - to preserve what works well in medicine and remake what does not. It is hard to imagine a more exciting time for our field.

## Box 2

**Thomas Powles**St Bartholomew's Hospital, London, UK

The field of medical oncology is moving at a breathtaking speed. A plethora of new agents are now available based on the molecular biology of specific tumors. The next step is to identify subsets of patients who benefit from therapy and move away from ‘one size fits all’ strategies. Therefore, biomarkers predicting response to these therapies are required. The application of whole genome sequencing, novel tracers within the context of functional imaging and circulating biomarkers, such as free circulating tumor DNA will be important pieces in this complex puzzle. There is also a need to develop therapies which focus on inducing longer remission rather than temporary disease controls. A collaborative international approach is required to achieve these goals.

## Evidence-based medicine: education, communication and collaboration

There has been increasing international focus on public health initiatives, development of healthcare policies and evidence-based guidelines to improve medical practice [38,39]. This is embedded in effective education strategies, which is evident from a continuing medical education intervention aimed at strengthening links between evidence-based and values-based medicine in healthcare personnel [40]. Researchers found this intervention led to improved values, such as openness to change, which are essential for improving medical care. Chris Del Mar (Box 3) recommends that going forward a more collaborative approach to decision-making between clinicians, patients and policy makers needs to be developed, and highlights the importance of transparency and communication.

There is also increasing focus on involving researchers based in low-to-middle income countries as principal investigators in local research projects. This is especially important as local knowledge helps to ask the ‘right’ questions in health research, ensures the best available evidence is accumulated and that all ethical aspects have been considered [41-43]. This is vital to guide healthcare policies and identify new tools and strategies; the consequences of not doing so is evident from a recent bibliometric analysis of childhood immunization research output from Africa. Since the onset of the Expanded Program on Immunization in 1974, vaccine research productivity in Africa has skewed toward those funded privately, with minimal research input from African authors, suggesting a need for better communication among all stakeholders [44]. Robert Snow points out (Box 4), conditions for research are now improving in Africa, and it is important that local researchers and governments work closely to drive the research output from these regions forward.

## Box 3

**Chris Del Mar**Bond University Gold Coast, Australia

Medicine will enter a new phase of concern about decreasing gains for increasing harms - including not just cost but also over-diagnosis and over-treatment. The medical profession has not been able hitherto to demonstrate an ability to make such cost-benefit decisions sensibly alone, and therefore there will be increasing input from society; increased demand for shared decision-making with the patient; and more directives from government. One important element of quality will soon be considered to be the extent to which the clinician has explicitly, clearly and carefully communicated the evidence in such a way that every patient is in a position to express a preference for the range of management options available. Evidence-based medicine will no longer be some hidden activity that clinicians may (or may not) engage in: it will become the currency expected for patient-clinician communication.

## Box 4

**Robert William Snow**Kenya Medical Research Institute, Kenya

Since I started work in Africa 30 years ago the landscape of science and research capacity has changed enormously. It is no longer legitimate to make excuses that model-based computing, laboratory science or gene sequencing can only be done in the north. The infrastructure and human capacity now exists in Africa to provide the best possible science for public health problems that face the continent. A fundamental requirement for any form of development is that countries have to take ownership of their problems. The next decade requires an active promotion by governments in Africa, and international partners that support regional development, of the expanding cohorts of African scientists who champion the very highest standards of medical and public health science within the region. Generating new research from within Africa holds untold promise. Unlike external research agendas and funding 30 years ago, this new research will have a much greater and much faster impact on the health of communities in Africa over the next decade.

## Enhancing research with reporting guidelines

Without clear guidelines for conducting and analyzing medical research, there is a limit to how far medicine can progress, and the last few years have seen many important improvements in reporting standards. In 2010, *BMC Medicine* co-published the updated CONSORT (CONsolidated Standards of Reporting Trials) statement by Schulz and colleagues [45]. This statement guides authors on the reporting of two-parallel design randomized controlled trials by using a checklist and flow diagram based on the latest methodological evidence. More recently, in response to the particular challenges in reporting economic evaluations of health interventions, the Consolidated Health Economic Evaluation Reporting Standards (**CHEERS**) was published [46]. This statement consolidates existing guidelines with the aim of providing more ‘user-friendly’ guidance for researchers and editors.

As research methods become more sophisticated, so too do the methods via which literature analysis can be conducted. The **‘RAMESES’** (Realist and Meta-review Evidence Synthesis: Evolving Standards) statement [47,48] was published to provide researchers, institutes and journals with guidance on how to conduct these new forms of literature analysis, and adherence to the guidelines will lead to quality assurance and uniform reporting of studies.

Ensuring consistency is a challenging task, and David Moher explains (Box 5) that journals and editors play a key role in providing peer reviewers and authors with the tools and guidance to ensure that medical research is appropriately reported.

We hope you have enjoyed our selection of just some of the most exciting content from *BMC Medicine,* and hope this has prompted you to seek out favorites of your own.

As an open access general medical journal, we aim to promote better informed clinical decisions and improved therapies. We will continue to publish content that has the potential to improve clinical practice, research and reporting. We especially encourage debate on health related issues not just within the clinical community, but also for the general public who should be, after all, the primary beneficiaries of the research.

## **Box 5**

**David Moher**Ottawa Hospital Research Institute, Canada

To reduce the considerable waste of inadequately published research, medical journals will need to develop long-term innovative strategies, such as developing core competencies for editors and peer reviewers, as well as accreditation programs for journals. More immediately, they can help foster greater implementation of reporting guidelines by facilitating the development of applications that can take manuscript content and automatically populate reporting guideline checklists. Such information can provide immediate feedback about the completeness of reporting of manuscripts to authors, editors and peer reviewers.

## Competing interests

Both authors are employees of BioMed Central, the publisher of *BMC Medicine*.

## Authors’ information

Sabina Alam is the Editor of *BMC Medicine*. Jigisha Patel is the Medical Editor at BioMed Central.
